# Prognostic and Predictive Value of Liquid Biopsy-Derived Androgen Receptor Variant 7 (AR-V7) in Prostate Cancer: A Systematic Review and Meta-Analysis

**DOI:** 10.3389/fonc.2022.868031

**Published:** 2022-03-18

**Authors:** Tanzila Khan, Therese M. Becker, Kieran F. Scott, Joseph Descallar, Paul de Souza, Wei Chua, Yafeng Ma

**Affiliations:** ^1^ School of Medicine, Western Sydney University, Campbelltown, NSW, Australia; ^2^ Medical Oncology, Ingham Institute of Applied Medical Research, Liverpool, NSW, Australia; ^3^ Centre of Circulating Tumour Cell Diagnostics & Research, Ingham Institute of Applied Medical Research, Liverpool, NSW, Australia; ^4^ South West Sydney Clinical School, University of New South Wales, Liverpool Hospital, Liverpool, NSW, Australia; ^5^ Ingham Institute of Applied Medical Research, Liverpool, NSW, Australia; ^6^ School of Medicine, University of New South Wales, Kensington, NSW, Australia; ^7^ Medical Oncology, Liverpool Hospital, Liverpool, NSW, Australia

**Keywords:** prostate cancer, AR-V7, liquid biopsy, prognosis, meta-analysis

## Abstract

In advanced prostate cancer, access to recent diagnostic tissue samples is restricted and this affects the analysis of the association of evolving biomarkers such as AR-V7 with metastatic castrate resistance. Liquid biopsies are emerging as alternative analytes. To clarify clinical value of AR-V7 detection from liquid biopsies, here we performed a meta-analysis on the prognostic and predictive value of androgen receptor variant 7 (AR-V7) detected from liquid biopsy for patients with prostate cancer (PC), three databases, the Embase, Medline, and Scopus were searched up to September 2021. A total of 37 studies were included. The effects of liquid biopsy AR-V7 status on overall survival (OS), radiographic progression-free survival (PFS), and prostate-specific antigen (PSA)-PFS were calculated with RevMan 5.3 software. AR-V7 positivity detected in liquid biopsy significantly associates with worse OS, PFS, and PSA-PFS (P <0.00001). A subgroup analysis of patients treated with androgen receptor signaling inhibitors (ARSi such as abiraterone and enzalutamide) showed a significant association of AR-V7 positivity with poorer OS, PFS, and PSA-PFS. A statistically significant association with OS was also found in taxane-treated patients (P = 0.04), but not for PFS (P = 0.21) or PSA-PFS (P = 0.93). For AR-V7 positive patients, taxane treatment has better OS outcomes than ARSi (P = 0.01). Study quality, publication bias and sensitivity analysis were integrated in the assessment. Our data show that liquid biopsy AR-V7 is a clinically useful biomarker that is associated with poor outcomes of ARSi-treated castrate resistant PC (CRPC) patients and thus has the potential to guide patient management and also to stratify patients for clinical trials. More studies on chemotherapy-treated patients are warranted.

**Systematic Review Registration:**

PROSPERO, CRD42021239353.

## Introduction

Prostate cancer (PC) is one of the most common male cancers. The androgen receptor (AR) pathway is critical in maintaining normal prostate tissue homeostasis, cancer development and progression (1). Therapies for PC include surgery and radiation for localized or early-stage cancer, while for advanced or metastatic PC, androgen deprivation therapy (ADT), with or without chemotherapy is the standard of care. However, patients eventually develop castration resistant PC (CRPC). Recent incorporation of novel androgen receptor signaling inhibitors (ARSi, e.g., enzalutamide (Enz), abiraterone (Abi)) and taxane-based chemotherapy have improved outcomes of CRPC patients over the past two decades (2).

Biomarkers detected in liquid biopsy (such as circulating tumor cells and cell-free tumor DNA) demonstrate good concordance with biomarkers detected in conventional tissue biopsy, especially for metastatic CRPC (3). Liquid biopsy is emerging as a reliable source of biological data for biomarker discovery, especially in advanced PC when tissue biopsy is often not obtainable or can be used longitudinally to monitor tumor evolution and changes in biomarker characteristics. In CRPC, one of most promising prognostic markers is the constitutively active AR splice variant 7 (AR-V7). AR-V7 lacks the ligand binding domain and substitutes for functional AR even in the absence of the ligand testosterone, and differentially regulates AR-dependent gene expression (4). Thus far, current literature suggests that expression or nuclear subcellular location of AR-V7 is associated with overall survival (OS) and progression free survival (PFS) when found in tissue biopsy (5) or liquid biopsy [whole blood (6, 7), circulating tumor cells (8), and exosomes (9, 10)]. However, the study cohorts are variable in patient numbers and stages and also treatment options; the clinical relevance of AR-V7, especially liquid biopsy detectable AR-V7, is still not clear or widely accepted and need further investigation.

To clarify the clinical utility of AR-V7 detection from liquid biopsies, we undertook a comprehensive systematic review and meta-analysis to evaluate the available data from the clinical studies published up to September 2021. Prognostic and predictive value of liquid biopsy derived AR-V7 data in PC patients were evaluated from 37 studies that met the inclusion criteria.

## Methods

### Study Design and Literature Searches

This study was conducted according to preferred reporting items for systematic reviews and meta-analysis (PRISMA) (11). The protocol has been registered on PROSPERO (CRD42021239353). Detailed literature searches up to September 10, 2021 in the Embase, PubMed, and Scopus databases were conducted thoroughly to check the prognostic role of AR-V7 in PC. The used search terms were (~Androgen Receptor Variant 7) OR (~ARV7) OR (~AR3) AND (~”prostate cancer”). The searched study citations were imported to EndNote (version X9) for duplicate checking and title and/or abstract screening and then uploaded to the online systematic review research tool Rayyan (https://www.rayyan.ai/) for independent systematic review according to selection criteria. Two independent, blinded observers (TK and YM) reviewed all candidate articles. Any discrepancies in the article selections were resolved by discussion.

### Selection Criteria

Pre-set exclusion criteria of this study were: (1) publication type: review articles, letters, comments, questionnaires, conference papers, corrections, reply to editor, case reports, book chapters, abstracts only, research highlights, summaries; (2) non-human studies (animal or cell line study); (3) non-prostate cancer; (4) AR-V7 data are not derived from human; (5) survival data not related to AR-V7 or with insufficient data to calculate the hazard ratios (HRs) and their 95% CIs, or the Kaplan–Meier (K–M) curve unable to calculate HRs and 95% CI parameters. Finally, studies were only included when they met the following criteria: (1) AR-V7 assayed in liquid biopsies (whole blood, circulating tumor cells, PBMC, plasma, exosome); (2) A reported relationship between AR-V7 and prognostic/predictive indicators, namely, OS, PFS, and PSA-PFS; (3) patient cohorts with n >25, and (4) English language only.

### Data Extraction and Quality Assessment

This study focuses on the prognostic value of AR-V7 detected from liquid biopsy and its predictive value for ARSi and chemotherapy. According to a pre-designed table, the items of data extraction included the last name of the first author, publication year, study country, number of patients included, age of patient, sample resource (processing method) and AR-V7 detection method, type of therapies, endpoints of oncological outcomes, HRs and 95% CIs (from univariate or multivariate Cox analysis), follow-up durations and definitions of OS, PFS, and PSA-PFS (**Supplementary Table 1**). When HRs and 95% CIs were not presented in the study, an Engauge Digitizer (version 12.1) was used to digitalize the K–M survival curve to re-calculate HRs and 95% CI as described previously (12). Data was extracted by two authors (TK and YM) independently and any inconsistencies were resolved by discussion. Notably, when several publications were retrieved reporting the same trial or patient cohort or from same author(s), study question and data from this publication were discussed by two authors (TK and YM) and uniqueness of the included data was ensured.

The adapted Newcastle–Ottawa Scale (NOS) scales for cohort study (13) were used to evaluate the quality of enrolled studies, which embraced three aspects, namely, patient selection, comparability, and assessment of outcome with a total score of 9. In addition, the quality of statistical evaluation was assessed to give a maximal score of 1 as described in **Supplementary Table 2**; a score of 7 or more is considered as high-quality and a score of 6 or less is considered as low quality.

### Statistical Analysis

Pooled HR and 95% CI were used to evaluate the prognostic and predictive value of AR-V7 presence or high expression (in some studies, authors set a threshold to discriminate high or low expression level) on the patient survival parameters (OS, PFS, PFA-PFS) in Review Manager 5.3 software (RevMan v.5.3, Denmark). The Cochran Q and I^2^ statistical methods were applied to evaluate the heterogeneity among included studies and a random effects model was used for data consolidation. If the heterogeneity was very high, only a descriptive score was given. Further subgroup analysis based on patient treatment was also conducted. The inverted funnel plots with Egger’s test were used to analyze potential publication bias with R software. A sensitivity analysis was carried out to assess the influence of each individual study on the pooled results by sequentially excluding each study. A two-tailed p-value <0.05 was regarded as statistically significant.

## Results

### Search Results, Study and Patient Characteristics

**Figure 1:**
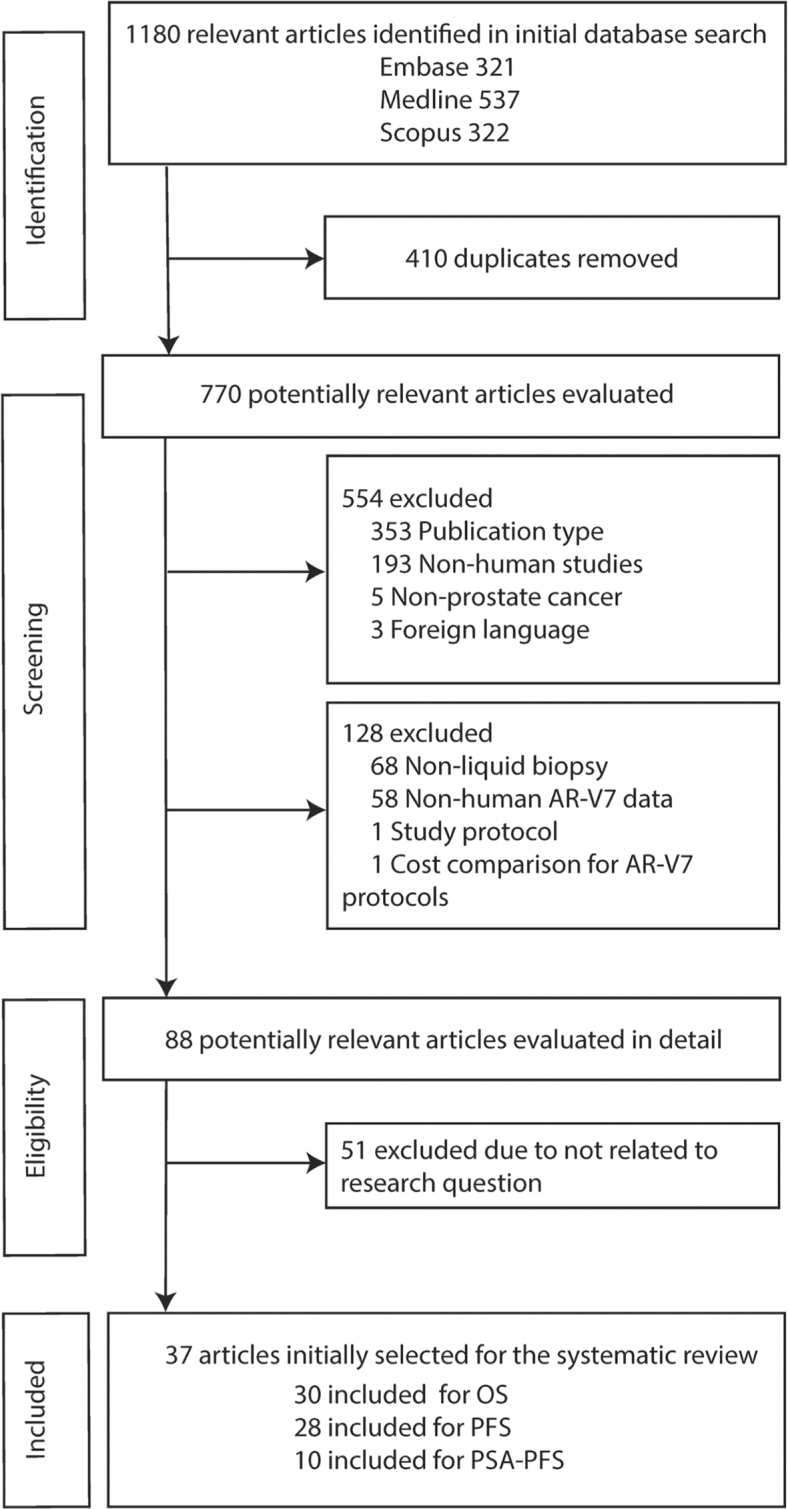
Flow chart of literature search and study selection.

The flowchart outlining the results of the literature search and application of the strategic inclusion and exclusion criteria is presented in **Figure 1**. A total of 1,180 relevant articles were identified in initial database searches (Embase: 321, Medline: 537, Scopus: 322). After screening research title and abstract to remove duplicates (n = 410) and excluding the non-relevant studies based on publication type (n = 353), non-human studies (n = 193), non-prostate cancer (n = 5) and foreign language (n = 3) followed by a review of full text for eligibility, 37 articles were identified based on inclusion criteria ‘human data’, ‘AR-V7’, ‘liquid biopsy’, and ‘survival’. Although we initially only searched quite a broad terminology ‘prostate cancer’, all 37 studies investigated CRPC (n = 4) or metastatic CRPC (mCRPC) (n = 33) as defined in the reports (**Supplementary Table 1**). Baseline characteristics of all eligible articles are listed in **Table 1**. All articles were published from 2014 to 2021 and included studies from Europe (46%), America and Canada (46%), and Asia-Pacific (8%). Liquid biopsy AR-V7 was detected from CTC (n = 28), PBMC (n = 2), whole blood (n = 4) or exosomes (n = 3). The patient cohort size ranged from 26 to 202 and the median or mean patient age ranged from 56 to 78. CTC enrichment methods included (modified) AdnaTest ^®^ (Qiagen) (n = 13), Oncoquick^®^ (Greiner Bio-One GmbH) (n = 1), red blood cell (RBC) lysis (n = 3), and immunomagnetic beads-based methods (such as CellSearch^®^ or IsoFlux^®^, dynabeads) (n = 9). The method of AR-V7 detection was primarily by PCR (quantitative PCR and droplet digital PCR, 92%). Endpoint of patient outcomes include OS (n = 30), PFS (n = 28) and PSA-PFS (n = 10) (**Table 1**).

**Table 1:** The basic characteristics of eligible studies.

Study	Year, country	Study type	Patients	Age	Resource, method	Treatment	Endpoint outcome	Follow up(month)	NOS score
**Antonarakis et al. (** 14 **)**	2015 US	Pros	37 CTC+	67 (46–82)^b^	CTCs (mAdna), qRT-PCR	Taxane	OS, PFS, PSA-PFS	7.7 (0.7–19.0)^b^	10
**Antonarakis et al. (** 15 **)**	2017 US	Pros	53 CTC−, 113 CTC+/AR-V7-, 36 CTC+/AR-V7+	70 71 70^a^	CTCs (mAdna), qRT-PCR	Abi/Enz	OS, PFS, PSA-PFS	CTC−:15.0 CTC+/ARV7-:21.7 CTC+/ARV7+:14.6^a^	9
**Antonarakis et al. (** 16 **)**	2014 US	Pros	Enz:31, Abi: 31	Enz:70 (56–84), Abi:69 (48–79)^b^	CTCs (mAdna), qRT-PCR	Abi/Enz	OS, r PFS, PSA-PFS	Enz: 5.4 (1.4–9.9) Abi: 4.6 (0.9–8.2)^b^	9
**Armstrong et al. (** 17 **)**	2019 US	Pros, blinded, multi-center	118	73 (45–92)^b^	CTCs (Adna, CellSearch), qRT-PCR	Abi/Enz	OS, PFS	19.6^a^	10
**Armstrong et al. (** 18 **)**	2020 US	Pros, blinded	ARSi:118 Taxane: 51	72 (48–82) 72 (45–87)^b^	CTCs (Adna, CellSearch), qRT-PCR	ARSi, Taxane	OS, PFS	ARSi:35 Tax:23^a^	9
**Belderbos et al. (** 19 **)**	2019 Netherlands	Pros	94	69 (65–75)^c^	CTCs (CellSearch), qRT-PCR)	Cabazitaxel ARSi	OS	NA	9
**Cattrini et al. (** 20 **)**	2019 Italy	Pros	39	72 (56–84)^b^	CTCs (Adna), qRT- PCR	ARSi, Taxane	OS	NA	8
**Chung et al. (** 21 **)**	2019 US	Pros	37	72 (67–79)^c^	CTCs (Dynabeads), qRT-PCR	Abi/Enz	OS, rPFS, PSA-PFS	11.4 (4.7–21.3)^c^	7
**De Laere et al. (** 22 **)**	2019 Belgium	Pros multi-center	168	76 ± 7.7^e^	CTCs (CellSearch), RNA-seq	Abi/Enz	OS, PFS	12.4 (7–17.3)^c^	10
**Del Re et al. (** 23 **)**	2017 Italy	Pros	36	66 (51–81)^b^	Plasma exosomes (exoRNeasy), ddPCR	ARSi	OS, PFS	9 (2.0–31.0)^b^	8
**Del Re et al. (** 9 **)**	2021 Italy	Retros	84	78 (47–91)^b^	Plasma exosomes (exoRNeasy), ddPCR	ARSi	OS, PFS	NA	9
**Del Re et al. (** 10 **)**	2019 Italy	Retros	73	NA	Plasma exosomes (exoRNeasy), ddPCR	Abi/Enz	OS, PFS	NA	7
**Erb et al. (** 24 **)**	2020 Germany	Pros	26	74.3 ± 9^a^	CTCs (OncoQuick), IHC	ARSi, Taxane	PFS	NA	6
**Graf et al. (** 25 **)**	2020 US	Pros, cross-sectional	193	69 (62.5–75)^c^	CTCs (RBC lysis), IF	ARSi, Taxane	OS	28.4 (24.4–33.0)^c^	9
**Gupta et al. (** 26 **)**	2019 US	Pros	ARSi:120 Radium:20	ARSi:73 (45–92) Radium:72 (54–86)^b^	CTCs (Adna, CellSearch), qRT-PCR and Epic assay	Abi/Enz, Radium	PFS	NA	9
**Joncas et al. (** 27 **)**	2019 Canada	Pros	35	75 (67,79)^c^	EVs (UC, miRNeasy), ddPCR	ARSi, Taxane	OS, PFS	27 (16,33)^c^	8
**Kwan et al. (** 28 **)**	2019 Australia	Pros	115	72 (46–91)^b^	WB, qRT-PCR	ARSi, Taxane	OS	15.5 (1.4–29)^b^	10
**Lorenzo et al. (** 29 **)**	2021 Italy	Pros, multi-center	53 (45 data only)	72.1 (54–86)^b^	CTCs, (Flow cytometry)	Enz	OS, rPFS	27^a^	10
**Maillet et al. (** 30 **)**	2019 France	Pros	41	73^a^	CTCs (AdnaTest), qRT-PCR	ARSi	OS, rPFS, PSA-PFS	31 ARSi treated patients: 10.5^a^	8
**Marín et al. (** 31 **)**	2020 Spain	Pros	136	ARSi:70.2 (53.3–93.3) Tax: 62.8 (32.8–79.4)^b^	PBMC and CTCs (IsoFlux) qRT-PCR	Abi/Enz, Taxane	OS, rPFS, PSA-PFS	ARSi:14.9 (1.5–57.9) Tax:13.8 (1.37–82.27)^a^	10
**Markowski et al. (** 32 **)**	2021 US	Multicohort phase II	Post-Abi: 29, Post-Enz: 30	Post-Abi: 71 (49–85) Post-Enz: 74 (50–89)^b^	CTCs (Adna), qRT-PCR	BAT, ARSi	rPFS	NA	7
**Miyamoto et al. (** 33 **)**	2018 US	Pros	27	67^d^	CTCs (CTC-iChip), ddPCR	Abi	OS, rPFS	13.0^a^	8
**Okegawa et al. (** 34 **)**	2018 Japan	Retros	49 CTC−, 23 CTC+/AR-V7−, 26 CTC+/AR-V7+	69 71 72^d^	CTCs (on-chip FC), PCR	Abi/Enz	OS, rPFS, PSA-PFS	20.7 (3.0–37.0)^b^	9
**Onstenk et al. (** 35 **)**	2015 Netherlands	Pros, multi-center, phase II	29	70 ± 7^e^	CTCs (CellSearch), qRT-PCR	Cabazitaxel	OS, PFS	7 (2–27)^b^	7
**Qu et al. (** 36 **)**	2017 US	Retros	Abi: 81, Enz: 51	Abi: 68.3 (62–74) Enz:69.0 (63–74)^c^	PBMC(Ficol), ddPCR	Abi/Enz	OS, PFS (TTF)	29.7 (3.6–47.5) 23.9 (0.9–48.3)^b^	10
**Scher et al. (** 37 **)**	2018 US	Pros, cross-sectional	142	69.5 ± 9.6^e^	CTCs (RBC lysis), IF	ARSi, Taxane	OS	4.3 years	8
**Scher et al. (** 38 **)**	2017 US	Pros, cross-sectional	161	68 (45–91)^b^	CTCs (RBC lysis), IF	ARSi, Taxane	OS	11 (1–30)^a^	9
**Scher et al. (** 39 **)**	2016 US	Pros, cross-sectional	161	68 (45–91)^b^	CTCs, IF	ARSi, Taxane	OS, PFS	36	10
**Seitz et al. (** 40 **)**	2017 Germany	Pros	85	71 (66–74)^c^	WB, ddPCR	Abi/Enz	OS, rPFS, PSA-PFS	7.6 (4.7–12.7)^c^	8
**Sepe et al. (** 41 **)**	2019 Italy	Pros	Abi:26, Enz: 11	75 (68–80)^b^	CTCs (Adna), qRT-PCR	Abi/Enz	OS, rPFS, PSA-PFS	25^a^	9
**Sharp et al. (** 8 **)**	2019 UK	Pros	181	CTC−:71.0 (66.8–75.6), CTC +/AR-V7−: 69.6 (64.9-72.3), CTC +/AR-V7−: 70.4 (65.3–74.6)^c^	CTCs (Adna, CellSearch), qRT-PCR	ARSi, Taxane	OS	19 (11–31)^c^	10
**Škereňová et al. (** 42 **)**	2018 Czech Republic	Retros	41	71 (54–82)^b^	CTCs (Adna), qRT-PCR	Docetaxel	OS	23.5^a^	7
**Stuopelyte et al. (** 6 **)**	2020 Lithuania	Pros	102	75.4 (11.4)^c^	WB, qRT-PCR	Abi	PFS, OS	30.5^a^	9
**Tagawa et al. (** 43 **)**	2019 US	Pros	54	71 (53–84)^b^	CTCs, ddPCR	Taxane	PFS	NA	7
**Todenhöfer et al. (** 7 **)**	2016, Canada	Pros	37	70 (53–87)^b^	WB, qRT-PCR	Abi	OS PSA-PFS	NA	8
**Tommasi et al. (** 44 **)**	2018 Italy	Pros	44	71.5 (55-87)^b^	CTCs (Adna), qRT-PCR	ARSi, Taxane	PFS	20.5^a^	7
**Wang et al. (** 45 **)**	2018 China	Pros	36	56.2 ± 8.6^e^	CTCs (immuno-beads), qRT-PCR	Abi/Enz	PFS	NA	6

Studies are labeled as last name of first author, et al. and presented in alphabetical order; Patient number and age are all patients included in study; Pros, prospective; Retros, retrospective. ^a^median, ^b^median (range), ^c^median IQR, ^d^mean, ^e^mean ± STD. WB, whole blood; CTC, circulating tumor cells; RBC, red blood cell lysis; PBMC, peripheral blood mononuclear cell; Ficoll, density gradient medium; Adna, AdnaTest ProstateCancerPanel AR-V7; mAdna, modified Adna; IF, immunofluorescent staining; qRT-PCR, quantitative real time-polymerase chain reaction; ddPCR, droplet digital PCR; UC, ultracentrifuge; FC, flow cytometry; ARSi, androgen receptor signaling inhibitor; Abi, abiraterone; Enz, Enzalutamide; BAT, bipolar androgen therapy; NA, not available; some studies include healthy control for threshold setting or discovery cohort (the data is lack and not included in table).

Thirty studies including 976 AR-V7 positive (or high level, as defined by authors) and 2,056 AR-V7 negative (or low level) patients were used for OS comparison, while 28 studies including 697 AR-V7 positive and 1,553 AR-V7 negative patients were used for PFS analysis and 10 studies including 216 AR-V7 positive and 425 AR-V7 negative patients for PSA-PFS analysis. Most patients in the cohort of studies were treated with ARSi (either enzalutamide, abiraterone, or not specified) or taxane-based chemotherapy. Some reports included miscellaneous treatments [such as Bipolar Androgen-based therapy (32)]. Overall AR-V7 positive patients had significantly worse OS (HR 3.36, 95% CI 2.56–4.41, P <0.00001), PFS (HR 2.96, 95% CI 2.20–3.98, P <0.00001) and PSA-PFS (HR 4.34, 95% CI 2.15–8.76, P <0.00001) than AR-V7 negative patients. Due to significant study heterogeneity (I^2^ ≥80%), random effects model was applied to calculate HR value and 95% CI for all survival parameters.

### Predictive Value of AR-V7 for ARSi-Treatment

**Figure 2:**
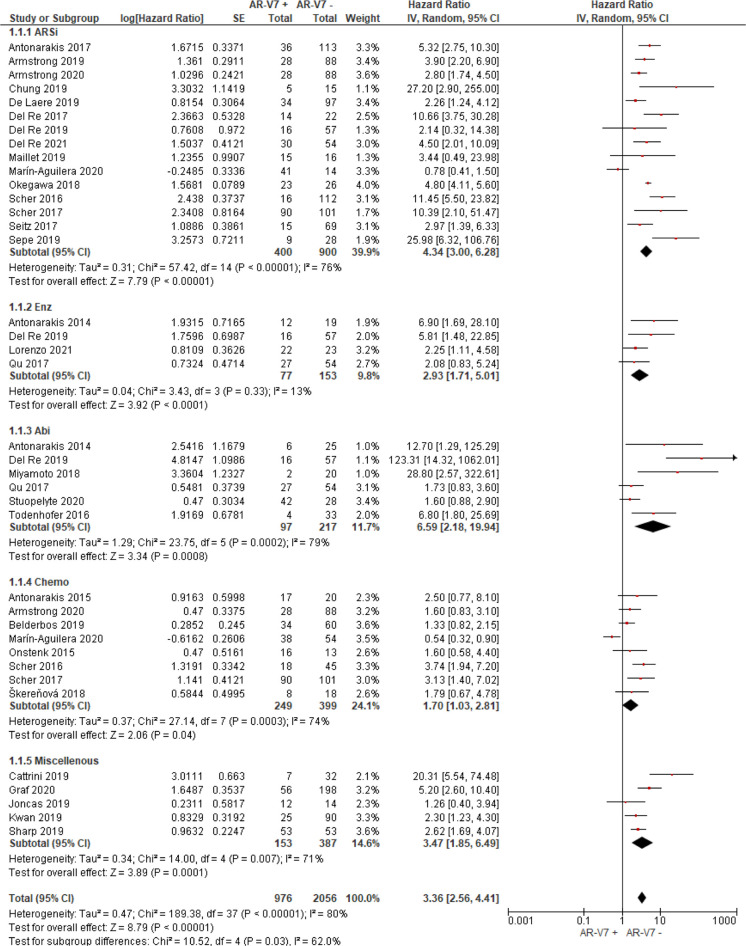
Forest plot of hazard ratios (HRs) for association of liquid biopsy AR-V7 status with overall survival (OS) in all included studies. Pooled HRs were calculated using random effect model. AR-V7, androgen receptor splice variant 7; CI, confidence interval and bars indicate 95% CIs. Subgroup analysis (ARSi, enzalutamide or abiraterone; Enz, enzalutamide; Abi, abiraterone; Chemo, taxane based chemotherapy; Miscellaneous, treatments that do not belong to above treatments or not clearly defined) were assessed.

**Figure 3:**
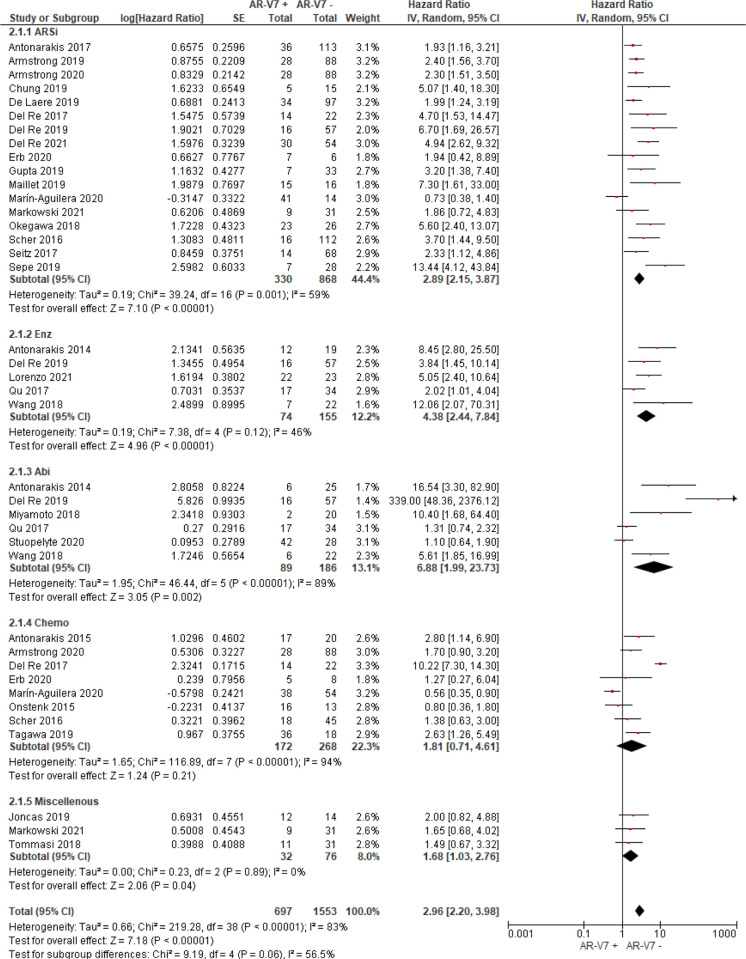
Forest plot of hazard ratios (HRs) for association of liquid biopsy AR-V7 status with PFS in all studies. Pooled HRs were calculated using random effect model. AR-V7: androgen receptor splice variant 7. CI, confidence interval and bars indicate 95% CIs. Subgroup analysis (ARSi, enzalutamide or abiraterone; Enz, enzalutamide; Abi, abiraterone; Chemo, taxane based chemotherapy; Miscellaneous, treatments that do not belong to above treatments or not clearly defined) were assessed.

**Figure 4:**
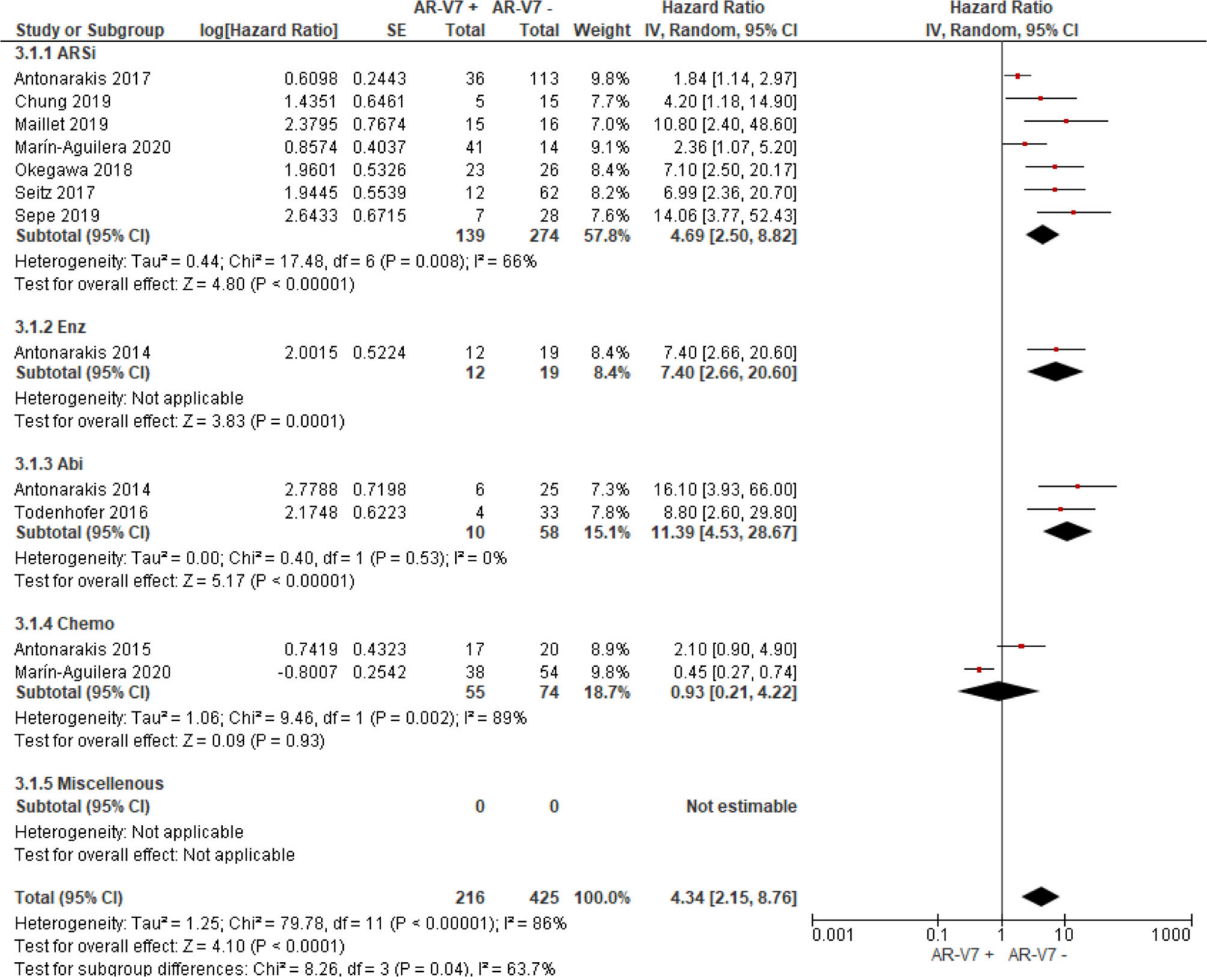
Forest plot of hazard ratios (HRs) for association of liquid biopsy AR-V7 status with PSA-PFS in all studies. Pooled HRs were calculated using random effect model. AR-V7, androgen receptor splice variant 7; CI, confidence interval and bars indicate 95% CIs. Subgroup analysis (ARSi, enzalutamide or abiraterone; Enz, enzalutamide; Abi, abiraterone; Chemo, taxane based chemotherapy; Miscellaneous, treatments that do not belong to above treatments or not clearly defined) were assessed.

AR-V7 positive patients treated with ARSi (enzalutamide or abiraterone) had significant poorer OS (HR 4.34, 95% CI 3.00–6.28, P <0.00001), PFS (HR 2.89, 95% CI 2.15–3.87, P <0.00001) and PSA-PFS (HR 4.69, 95% CI 2.50–8.82, P <0.0001) compared with AR-V7 negative patients (**Figures 2** **–** **4**). When analyzed based on specific treatment, compared to negative patients, AR-V7 positive patients also had significant worse OS (Enz: HR 2.93, 95% CI 1.71–5.01, P <0.0001; Abi: HR 6.59, 95% CI 2.18–19.94, P = 0.0008, respectively) (**Figure 2**), PFS (Enz: HR 4.38, 95% CI 2.44–7.84, P <0.0001; Abi: HR 6.88, 95% CI 1.99–23.73, P = 0.002, respectively) (**Figure 3**) and PSA-PFS (Enz: HR 7.40, 95% CI 2.66–20.60, one study, P = 0.0008; Abi: HR 11.39, 95% CI 4.53–28.67, two studies, P <0.00001, respectively) (**Figure 4**).

### Chemotherapy-Treated Patients and Outcome Association With AR-V7

In the subgroup analysis of the patients treated with taxane-based chemotherapy, the association of AR-V7 positivity with worse OS was observed (HR 1.70, 95% CI 1.03–2.81, P = 0.04) (**Figure 2**), but no conclusive association between AR-V7 positive status and worse PFS and PSA-PFS were apparent, likely due to inadequate power (PFS: HR 1.81, 95% CI 0.71–4.61, P = 0.21, **Figure 3**; PSA-PFS: HR 0.93, 95% CI 0.21–4.22, P = 0.93, **Figure 4**). It is to be emphasised that data is only derived from two studies and a total of 129 patients (**Figure 4**).

### AR-V7 Effect on Non-Defined (Miscellaneous) Treatments

For the studies in which the authors did not clarify treatments and were unable to be classified as either ARSi or taxane chemotherapy, AR-V7 presence is associated with worse OS (HR 3.47, 95% CI 1.85–6.49, P = 0.0001, 5 studies) and PFS (3 studies, HR 1.68, 95% CI 1.03–2.76, P = 0.04) (**Figures 2**, **3**).

### ARSi vs. Chemotherapy in AR-V7 Positive or Negative Patients

**Figure 5:**
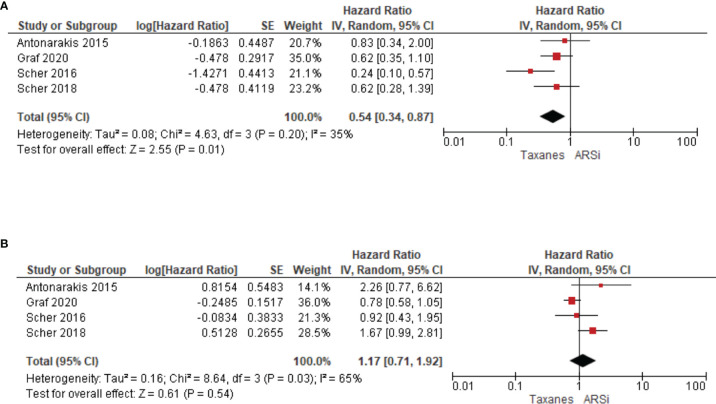
Forest plots for association of liquid biopsy AR-V7 status with OS in **(A)** AR-V7 positive (ARSi vs. Chemotherapy) and **(B)** AR-V7 negative patients (ARSi vs. Chemotherapy). Pooled HRs were calculated using random effect model. AR-V7, androgen receptor splice variant 7; CI, confidence interval and bars indicate 95% CIs.

Four studies compared treatment response in AR-V7 positive or negative patients. Taxane treatment is linked to superior OS (HR 0.54, 95% CI 0.34–0.87, P = 0.01) in patients positive for AR-V7, compared to ARSi (**Figure 5A**). In contrast, for AR-V7 negative patients, OS in taxane or ARSi treated patients is not significantly different (HR 1.17, 95% CI 0.71–1.92, P = 0.54) (**Figure 5B**).

### Quality Assessment, Publication Bias and Sensitivity Analysis

Thirty five articles were assessed as high-quality studies while 2 were deemed low quality studies (**Table 1** and **Supplementary Table 2**). Overall, the average quality of studies is 8.5. Publication biases were evaluated for subgroups with more than 10 publications; no publication bias was observed for OS (Egger’s test P = 0.9925, 15 publications, **Supplementary Figure 1A**) whereas publication bias was observed for PFS (Egger’s test P = 0.0411, 17 publications, **Supplementary Figure 1B**) in ARSi-treated subgroups. Sensitivity analyses were performed on the subgroups of more than 6 studies and the results were relatively stable except for overall survival in chemotherapy-treated group, where missing data in one study (31) had a significant effect on data outcome (**Supplementary Table 3**).

## Discussion

AR splice variants have been proposed as a cause of resistance to ARSi and taxane-based chemotherapy (46). AR-V7, the most-studied AR splice variant, is emerging as a clinically relevant biomarker in CRPC, with a detection incidence ranging between 20 and 60%, depending on biopsy source, detection methods, and disease stage. Given that tumor tissue of advanced PC is rarely available and archival tissue may not reflect the biology of the current tumor stage, liquid biopsies, mainly blood, are becoming attractive resources for AR-V7 and other biomarker evaluation. Technical advances, different detection methods for AR-V7 from liquid biopsies are now available, including modified AdnaTest ProstateCancer, and droplet digital PCR of CTCs enriched by various CTC isolation platforms (see **Table 1**). We recently confirmed CTC-based AR-V7 testing is more reliable than exosomal RNA and cell free tumor RNA in plasma (47). Accumulating reports on the association of AR-V7 detectability in liquid biopsy with therapy response and patient survival have prompted us to perform this systematic review and meta-analysis on the prognostic and predictive utility of liquid biopsy-based AR-V7 identification. Our data show that liquid biopsy detectable AR-V7 significantly associates with poor outcomes to ARSi treatment as shown for OS, PFS, PSA-PFS (P <0.001). This strongly supports the notion that AR-V7 detection from CRPC patient liquid biopsies has prognostic and predictive power. This observation is highly clinically relevant and could affect how clinicians make treatment decisions for patients with (metastatic) CRPC and when to transition patients to taxane-based chemotherapy.

While on taxane-based treatment, the association of AR-V7 presence with poorer outcome is still significant (P = 0.04) for OS data and lack adequate power for PFS (P = 0.21) or PSA-PFS (P = 0.93). However, there are relatively fewer publications in this subgroup, so these conclusions are based on weaker datasets compared to the ARSi treated subgroup; for instance, the omitting one publication changes the P-value and AR-V7 impact on OS would no longer be significant (**Supplementary Table 3**). Our data agree with a recent report that AR-V7 may contribute to taxane resistance by circumventing taxane-induced inhibitory effects both *in vitro* (cell lines) and *in vivo* (PC tissue) (43, 48). On the other hand, we cannot exclude the possibility that AR-V7 expression was induced in CRPC patients who had received ARSi prior to chemotherapy, and that its effect on OS has not been completely washed out by taxanes. We note that four studies suggest that chemotherapy would be a better option compared to ARSi (HR 0.54, P = 0.01) in AR-V7 positive CRPC, suggesting that AR-V7 determination is important in chemotherapy-treated patients. More studies in this subgroup are warranted.

Three other meta-analyses on AR-V7 prognostication (13, 49, 50) have been published recently, but given the common inaccessibility of current tissue biopsies, our meta-analysis exclusively focuses on liquid biopsies and includes the most up-to-date studies. Further, we not only include all studies with author self-reported HR and 95% CI, but also calculate HR and 95% CI with established methods (12) for some papers with insufficient and incomplete statistical reporting. Nevertheless, our systematic review has limitations. We only examined OS, PFS, and PSA-PFS, and did not assess other treatment outcomes such as PSA response. Discrepancies in the definition of PSA response (e.g., extent of PSA fall in a specific timeframe) exist across studies and given our selection criteria, papers were excluded if they only reported PSA response without survival data. Secondly, statistical power was limited by the numbers of studies available and small sample sizes in some of the subgroups analysed. Thirdly, included study designs differed greatly in biological material investigated (type of liquid biopsy and content such as CTCs or exosomes). For some studies, patients were enrolled from a single centre, potentially leading to publication bias and selection bias. Also, no randomized study has ever directly compared the predictive value of AR-V7 in patients treated with chemotherapy vs. ARSi. Therefore, the results are indirect with potential bias. Lastly, the variability of techniques used to determine AR-V7 positivity, namely, qRT-PCR and ddPCR of mRNA derived from CTC, whole blood, exosome, could result in differing conclusions. The cut-off value is essential in defining and interpretation of AR-V7 positivity, due to the continuous nature of this variable; more work is required to answer the question of whether the degree of AR-V7 presence is important. Last but not least, other CTC AR detection methods have been adopted such as RNA-seq and immunostaining. Despite the variety of methodologies, we found that liquid biopsy detectable AR-V7 correlates with disease outcomes (**Supplementary Figure 2**).

In conclusion, ARSi and taxane-based chemotherapy are approved treatment options for CPRC patients and are used globally. Use of emerging methodologies, such as liquid biopsy-determined AR-V7, to optimize utility of a known predictive biomarker could help to guide the optimal treatment sequencing pathway for each patient in a personalised manner and is therefore of clinical importance. Standardization of liquid biopsy AR-V7 detection would underpin utility in clinical practice. Avoiding ineffective therapies or early switching to more effective approaches should ensure better outcomes for patients. However, further studies on chemotherapy-treated patient cohort and direct comparison of chemotherapy vs. ARSi are warranted.

## Data Availability Statement

The original contributions presented in the study are included in the article/**Supplementary Material**. Further inquiries can be directed to the corresponding author.

## Author Contributions

Project development, methodology, data collection and analysis: TK and YM. Conceptualization: YM and TK. Project development: TB, KS, PDS and WC. Statistics: JD, TK and YM. Manuscript writing, editing, and reviewing: all authors. All authors listed have made a substantial, direct, and intellectual contribution to the work and approved it for publication.

## Funding

TK received an Ingham Institute/Narellan Rotary Club Men’s health grant 2018 and a WSU School of Medicine Androgen Receptor Research scholarship.

## Conflict of Interest

The authors declare that the research was conducted in the absence of any commercial or financial relationships that could be construed as a potential conflict of interest.

## Publisher’s Note

All claims expressed in this article are solely those of the authors and do not necessarily represent those of their affiliated organizations, or those of the publisher, the editors and the reviewers. Any product that may be evaluated in this article, or claim that may be made by its manufacturer, is not guaranteed or endorsed by the publisher.

## Supplementary Material

Supplementary Table 2 Quality assessment of included studies based on adapted NOS scales.

Supplementary Table 3 Sensitivity analysis of subgroups with more than 6 studies.

Supplementary Figure 1 Inverted funnel plot to evaluate potential publication bias in OS **(A)** and PFS **(B)** of ARSi treated patients.

Supplementary Figure 2 Forest plot of hazard ratios (HRs) for association of liquid biopsy AR-V7 status with OS **(A)**, PFS **(B)**, PSA-PFS **(C)** in all studies. Subgroup analysis were performed based on AR-V7 detection technique type. Pooled HRs were calculated using random effect model. AR-V7: androgen receptor splice variant 7. CI: confidence interval and bars indicate 95% CIs. PCR, polymerase chain reaction; qRT-PCR, quantitative real time PCR; ddPCR, droplet digital PCR; IF, immunofluorescence; IHC, immunohistochemistry; FC, flow cytometry; RNA-seq, RNA-sequencing.
