# Supplementary figures and images for: Prognostic and Predictive Value of Liquid Biopsy-Derived Androgen Receptor Variant 7 (AR-V7) in Prostate Cancer: A Systematic Review and Meta-Analysis
Source: Front Oncol. 2022 Mar 18;12:868031. doi: 10.3389/fonc.2022.868031 (PMC8971301; doi:10.3389/fonc.2022.868031)

A

Standard Error

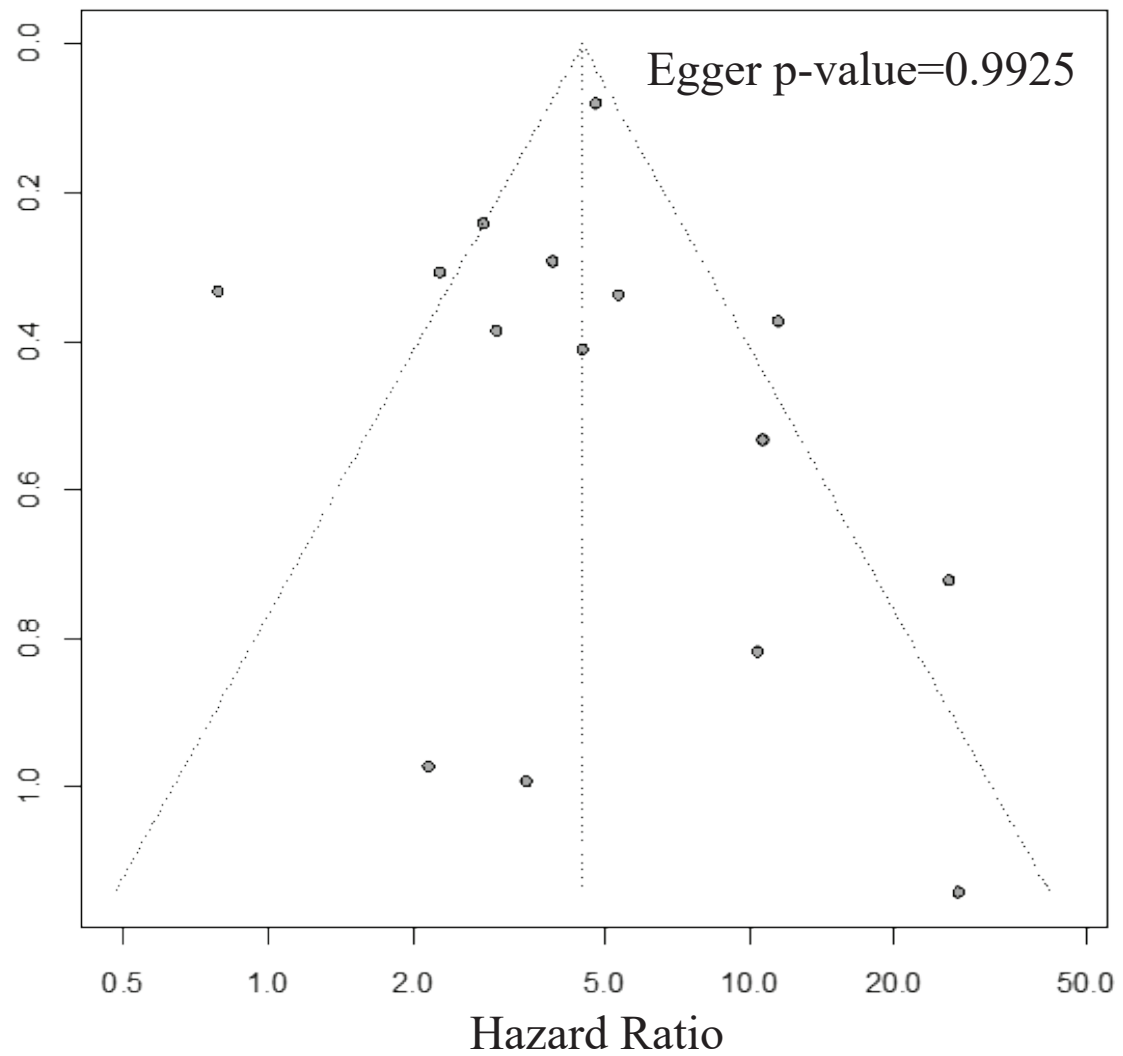

B

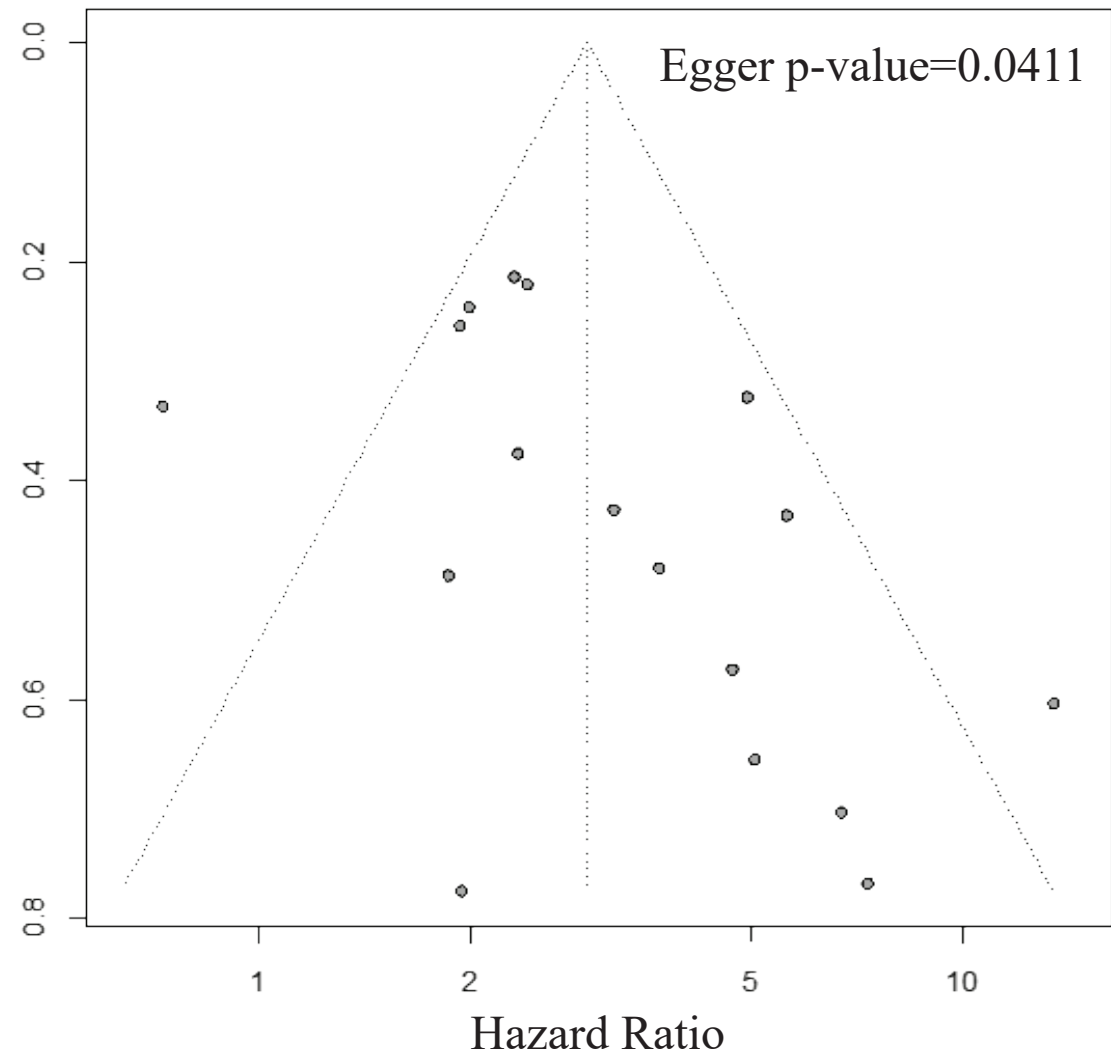

Supplement: Supplementary file 1 [file DataSheet_1.pdf]

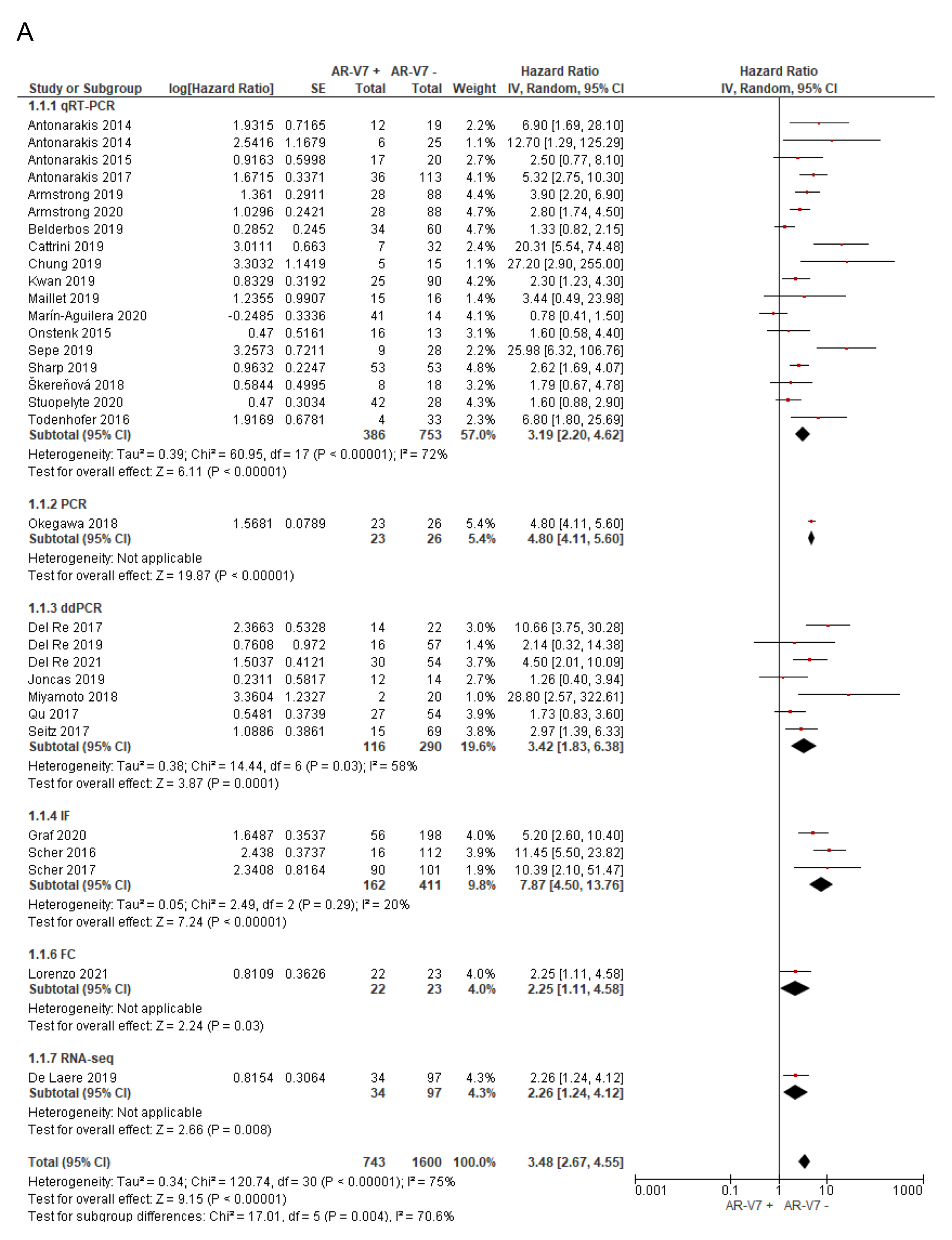

Supplement: Supplementary Figure 1 — Inverted funnel plot to evaluate potential publication bias in OS (A) and PFS (B) of ARSi treated patients. [file Image_1.tiff]

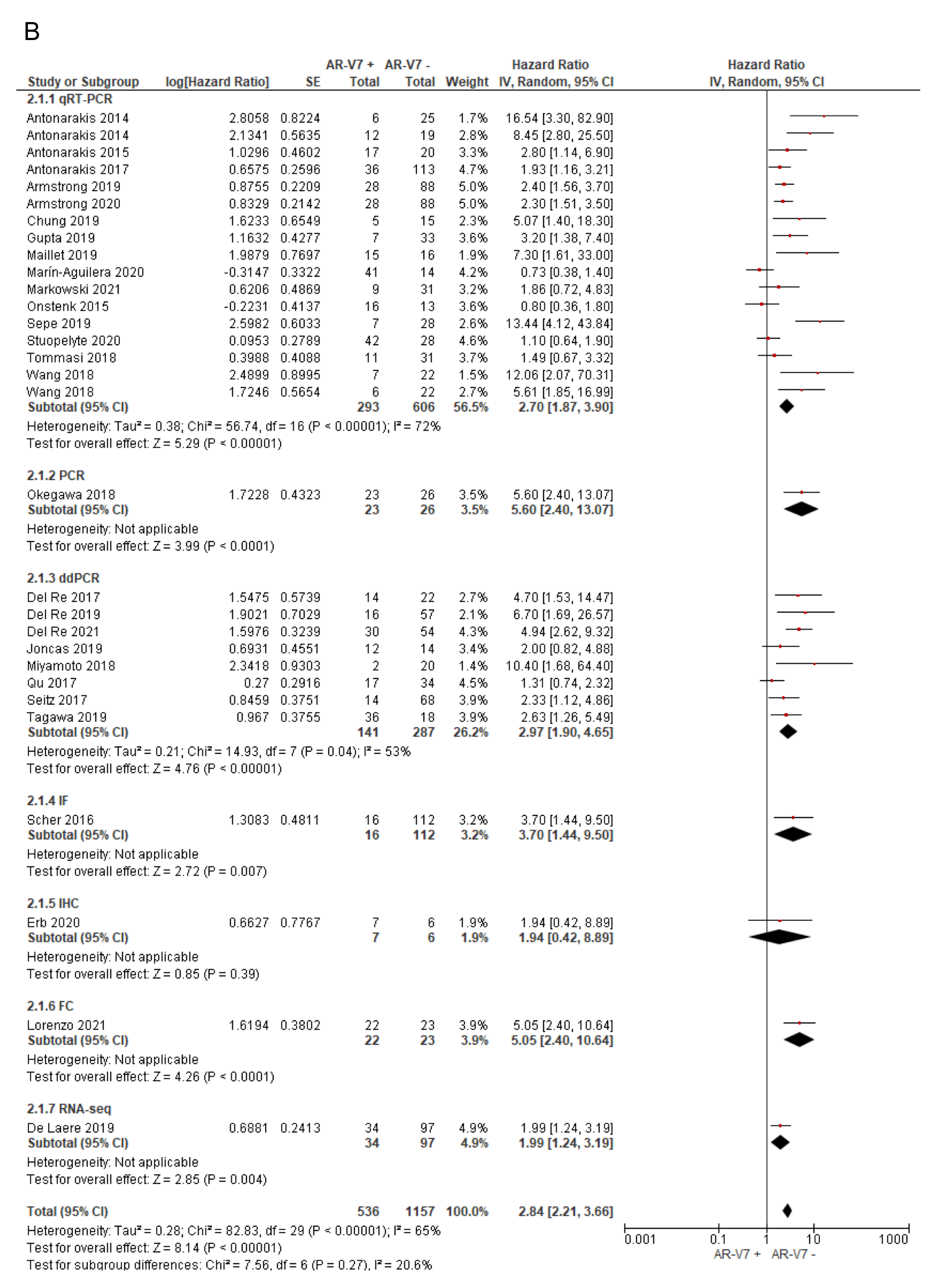

Supplement: Supplementary Figure 2 — Forest plot of hazard ratios (HRs) for association of liquid biopsy AR-V7 status with OS (A), PFS (B), PSA-PFS (C) in all studies. Subgroup analysis were performed based on AR-V7 detection technique type. Pooled HRs were calculated using random effect model. AR-V7: androgen receptor splice variant 7. CI: confidence interval and bars indicate 95% CIs. PCR, polymerase chain reaction; qRT-PCR, quantitative real time PCR; ddPCR, droplet digital PCR; IF, immunofluorescence; IHC, immunohistochemistry; FC, flow cytometry; RNA-seq, RNA-sequencing. [file Image_2.tiff]

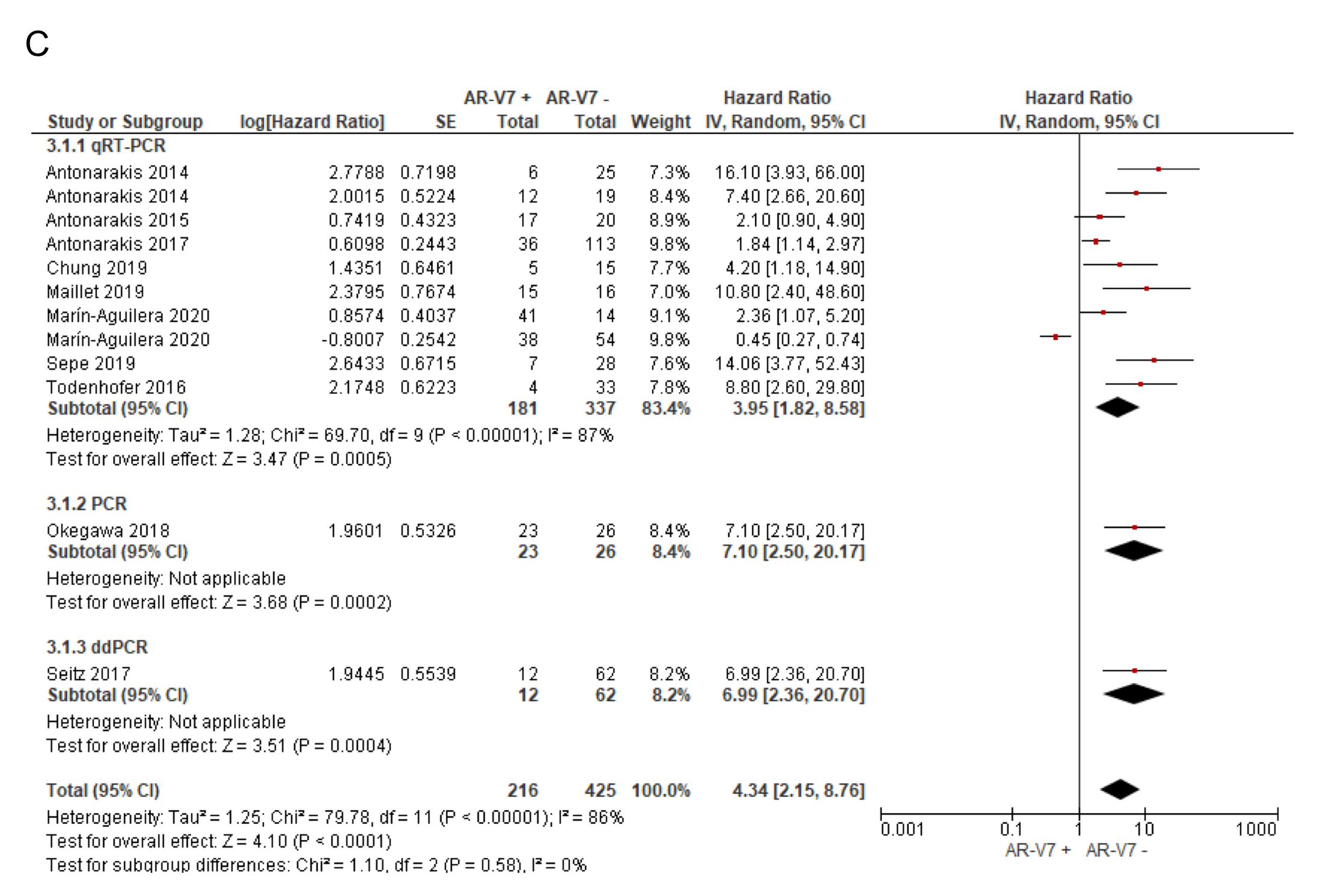

Supplement: Supplementary file 7 [file Image_3.tiff]
