# Supplementary material for: Prognostic and Predictive Value of Liquid Biopsy-Derived Androgen Receptor Variant 7 (AR-V7) in Prostate Cancer: A Systematic Review and Meta-Analysis
Source: Front Oncol. 2022 Mar 18;12:868031. doi: 10.3389/fonc.2022.868031 (PMC8971301; doi:10.3389/fonc.2022.868031)
Supplement: Supplementary file 2 [file Table_1.docx]

**Supplemental table1: information on definitions of OS, PFS, PSA-PFS and tumour stage in each included study. NA: Not available**

| Study | Stage | Outcome | OS | PFS | PSA-PFS |
| --- | --- | --- | --- | --- | --- |
| Antonarakis  et al 2015 | mCRPC | OS, PFS,  PSA-PFS | the time to death from any cause | symptomatic progression and radiologic progression or death, whichever occurred first | NA |
| Antonarakis  et al 2017 | mCRPC | OS, PFS,  PSA-PFS | the interval from enrolment to death from any cause | Same as above | NA |
| Antonarakis et al 2014 | mCRPC | OS, PFS,  PSA-PFS | Same as above in 1 | Same as above in 1 | NA |
| Armstrong  et al 2019 | mCRPC | OS, PFS | NA | from date of registration to clinical/radiographic progression or death, whichever occurred first |  |
| Armstrong  et al 2020 | mCRPC | OS, PFS | NA | Same as above in 4 |  |
| Belderbos  et al 2019 | mCRPC | OS | The date from enrolment to death from any cause |  |  |
| Cattrini  et al 2019 | mCRPC | OS | the time elapsed from blood collection date and the date of death for any cause |  |  |
| Chung  et al 2019 | mCRPC | OS, rPFS,  PSA-PFS | NA | a ≥20% increase in the sum of the soft tissue lesion diameters during computed tomography, ≫2 new bone lesions on nuclear medicine bone scan, or symptomatic progression (pain aggravation or cancer-related complications) | PSA progression was defined using the Prostate Cancer Working Group 3 definition as a ≥25% increase in PSA levels above the nadir (and by ≫2 ng/ml), with confirmation ≥4 weeks later |
| De Laere  et al 2019 | mCRPC | OS, PFS | NA | according to Prostate Cancer Clinical Trials Working Group 3 criteria |  |
| Del Re  et al 2017 | mCRPC | OS, PFS |  | Patients must have had at least three increasing serum PSA values taken at least 2 wk before the last value of at least 2.0 ng/ml, consistent with the Prostate Cancer Working Group-2 guidelines |  |
| Del Re  et al 2021 | mCRPC | OS, PFS |  | according to Prostate Cancer Clinical Trials Working Group 3 (PCWG3) guidelines |  |
| Del Re  et al 2019 | mCRPC | OS, PFS |  | the Prostate Cancer Working Group-2 guidelines |  |
| Erb  et al 2020 | mCRPC | PFS |  |  |  |
| Graf  et al 2020 | mCRPC | OS | from the time of treatment decision to death |  |  |
| Gupta  et al 2019 | mCRPC | PFS |  | the date from registration to radiographic progression using PCWG2 criteria, clinical progression requiring a change in systemic therapy, or death, whichever came first |  |
| Joncas  et al 2019 | CRPC | OS, PFS | NA | NA |  |
| Kwan  et al 2019 | mCRPC | OS | time from systemic treatment commencement to death from any cause |  |  |
| Lorenzo  et al 2021 | mCRPC | OS, rPFS | the time from the date of the enrolment to the date of death due to any cause | the time from enrolment to radiographic progressive disease or death due to any cause |  |
| Maillet  et al 2019 | mCRPC | OS, rPFS,  PSA-PFS | NA | Radiological progression was defined using PCWG3 criteria | PSA progression was defined using PCWG3 criteria as a post-treatment PSA level increase of >25% above the nadir, which is confirmed by a second value 3 wk later and a PSA measurement of 2ng/ ml |
| Marín  et al 2020 | mCRPC | OS, r-PFS,  PSA-PFS | calculated from the date of treatment initiation to death or last follow-up visit | calculated from the date of treatment initiation to RX progression | calculated from the date of treatment initiation to date of progression |
| Markowski et al 2021 | mCRPC | rPFS |  | Clinical or radiographic progression was defined by RECIST 1.1 (soft tissue lesions) and PCWG2 (clinical and bone lesions) |  |
| Miyamoto  et al 2018 | mCRPC | OS, rPFS | the interval between the start of therapy and the date of death or censor | the interval between the start of therapy and the date of radiographic progression, death, or censor |  |
| Okegawa  et al 2018 | mCRPC | OS, rPFS,  PSA-PFS | NA | determined by independent blinded review of available radionuclide bone scans, CT, or MRI, using the PCWG2 criteria (rPFS was defined as ≥2 new lesions on an 8-week bone scan plus two additional lesions on a confirmatory scan, ≥2 new confirmed lesions on any scan ≥12 weeks after random assignment, progression in nodes or viscera on cross-sectional imaging, or death.) | PSA progression was determined by PSAWG2; a patient was considered as experiencing biochemical failure if their PSA post-treatment determination increased by 50% and PSA measurement was ≥2 ng/mL |
| Onstenk  et al 2015 | mCRPC | OS, PFS |  | Reported end points were based on the Prostate Cancer Working Group 2 guidelines |  |
| Qu  et al 2017 | CRPC | OS, PFS (TTF) | time from treatment initiation to death from any cause, censored at the date of last follow-up for patients who were still alive | time from treatment initiation until the date of drug discontinuation for any reason, censored at the date of last follow-up for patients who were still on therapy |  |
| Scher  et al 2018 | mCRPC | OS | NA |  |  |
| Scher  et al 2017 | mCRPC | OS | calculated from initiation of therapy to death from any cause, with right-censoring for patients alive at last follow-up |  |  |
| Scher  et al 2016 | mCRPC | OS, PFS | calculated from initiation of therapy to death from any cause. Patients still alive at time of last follow-up were right-censored | Radiographic progression was determined by independent blinded review of available radionuclide bone scans, CTs, or MRIs, using the PCWG2 criteria,17 and calculated from therapy initiation until radiologically confirmed progression or death owing to any cause within 60 days of stopping treatment. Patients without evidence of radiologic progression at the time of last stable scan or end of therapy, whichever occurred later, were right censored |  |
| Seitz  et al 2017 | mCRPC | OS, rPFS,  PSA-PFS | NA | Clinical progression was defined as worsening of disease related symptoms or new cancer-related complications, radiographic progression according to Response Evaluation Criteria In Solid Tumors, two or more new bone lesions on bone scan, or death, whichever occurred first | PSA progression-free survival (PSA-PFS) according to PCWG2 criteria |
| Sepe  et al 2019 | mCRPC | OS, rPFS,  PSA-PFS | the time from the date of the start of treatment to death from any cause | defined as the time from the start of treatment to the first objective evidence of radiographic disease progression | defined as freedom time from PSA progression |
| Sharp  et al 2019 | mCRPC | OS | defined as time from PB draw to date of death or last follow up/contact |  |  |
| Škereňová  et al 2018 | mCRPC | OS | NA |  |  |
| Stuopelyte  et al 2020 | CRPC | PFS,  OS | the time from the initiation of the AA treatment until death from any cause | the time from the initiation of the AA treatment until documented evidence of disease progression |  |
| Tagawa  et al 2019 | mCRPC | PFS |  | the time between randomization and the first documentation of radiographic tumor progression (using RECIST 1.1), clinical progression (including skeletal-related events, increasing pain requiring escalation of narcotic analgesics, urinary obstruction, etc.), PSA progression, or death from any cause. PFS was required to be confirmed at least 3 weeks after initial assessment |  |
| Todenhöfer et al 2016 | mCRPC | OS  PSA-PFS | NA |  | PSA progression according to Prostate Cancer Working Group 2 criteria |
| Tommasi  et al 2018 | mCRPC | PFS |  | NA |  |
| Wang  et al 2018 | CRPC | PFS |  | NA |  |
